# Damage-free Metal Electrode Transfer to Monolayer Organic Single Crystalline Thin Films

**DOI:** 10.1038/s41598-020-61536-8

**Published:** 2020-03-13

**Authors:** Tatsuyuki Makita, Akifumi Yamamura, Junto Tsurumi, Shohei Kumagai, Tadanori Kurosawa, Toshihiro Okamoto, Mari Sasaki, Shun Watanabe, Jun Takeya

**Affiliations:** 1 Material Innovation Research Center (MIRC) and Department of Advanced Materials Science, Graduate School of Frontier Sciences, The University of Tokyo, 5-1-5 Kashiwanoha, Kashiwa, Chiba, 277-8561 Japan; 2 AIST-UTokyo Advanced Operando-Measurement Technology Open Innovation Laboratory (OPERANDO-OIL), National Institute of Advanced Industrial Science and Technology (AIST), 5-1-5 Kashiwanoha, Kashiwa, Chiba, 277-8561 Japan; 3 International Center for Materials Nanoarchitectonics (WPI-MANA), National Institute for Materials Science (NIMS), 1-1 Namiki, Tsukuba, 305-0044 Japan; 4 PRESTO, JST, 4-1-8 Honcho, Kawaguchi, Saitama, 332-0012 Japan

**Keywords:** Electronic devices, Organic molecules in materials science, Surface patterning

## Abstract

Solution-processed organic thin film transistors (OTFTs) are an essential building block for next-generation printed electronic devices. Organic semiconductors (OSCs) that can spontaneously form a molecular assembly play a vital role in the fabrication of OTFTs. OTFT fabrication processes consist of sequential deposition of functional layers, which inherently brings significant difficulties in realizing ideal properties because underlayers are likely to be damaged by application of subsequent layers. These difficulties are particularly prominent when forming metal contact electrodes directly on an OSC surface, due to thermal damage during vacuum evaporation and the effect of solvents during subsequent photolithography. In this work, we demonstrate a simple and facile technique to transfer contact electrodes to ultrathin OSC films and form an ideal metal/OSC interface. Photolithographically defined metal electrodes are transferred and laminated using a polymeric bilayer thin film. One layer is a thick sacrificial polymer film that makes the overall film easier to handle and is water-soluble for dissolution later. The other is a thin buffer film that helps the template adhere to a substrate electrostatically. The present technique does not induce any fatal damage in the substrate OSC layers, which leads to successful fabrication of OTFTs composed of monolayer OSC films with a mobility of higher than 10 cm^2^ V^−1^ s^−1^, a subthreshold swing of less than 100 mV decade^−1^, and a low contact resistance of 175 Ω⋅cm. The reproducibility of efficient contact fabrication was confirmed by the operation of a 10 × 10 array of monolayer OTFTs. The technique developed here constitutes a key step forward not only for practical OTFT fabrication but also potentially for all existing vertically stacked organic devices, such as light-emitting diodes and solar cells.

## Introduction

Organic semiconductors (OSCs) can spontaneously form a molecular assembly from a solution processed near room temperature, which makes them a promising material for next-generation electronic devices such as flexible displays^1–3^, low-cost radio-frequency identification tags^4,5^, and wearable sensing devices^6–9^. Recent intensive studies on synthetic chemistry and device engineering have led to the development of OSC compounds with a high carrier mobility of >10 cm^2^ V^−1^ s^−1^ combined with sufficient environmental stability^10–13^ and improved fabrication techniques that can produce large crystalline thin films of OSCs with areal coverages up to 100 cm^2^ ^10,14–19^. The unique functionalities and excellent processability of OSCs allow the ideal production of highly integrated electronic circuits based on organic thin-film transistors (OTFTs).

Generally, OTFT processes require sequential deposition of functional components, such as gate electrodes, gate dielectrics, active OSCs, and contact electrodes. This approach inevitably faces the serious issue that underlayers are affected by subsequent processes, and it is considerably problematic when fabricating top-contact OTFTs because OSC layers are susceptible to thermal damage during vacuum evaporation of metal electrodes^20–23^, as well as during the following photolithography processes^24,25^.

A photolithography process is well-established method to obtain precisely patterned electrodes. However, to employ photolithography process to form contact electrodes on the surface of OSC films, extra cares should be taken because of their poor durability against photoresist, and developer. Regarding this issue, a lift-off process which utilizes a protective layer has been proposed to avoid the damage from photoresist and developer on OSC layers^25^. This process allows a precise patterning of electrodes on versatile OSC materials, however, there still remains the problem of damages on OSC layers originated from the vacuum evaporation of metallic contacts. The quality of the metal/OSC interface is known to deteriorate significantly due to uncontrollable thermal diffusion of metal nanoparticles into the OSC layer^20,21^ and due to simple radiation damage^22,23^. Because this interface dominates carrier injection properties as well as interfacial contact resistance, an abrupt, heterogeneous interface needs to be established.

Forming an electrode on the surface of an OSC layer becomes more challenging when ultra-thin molecular layers are used as an active semiconductor. Much research has been conducted on making highly ordered crystalline films with a thickness of only a few molecular layers^14,26,27^. These ultrathin films can be expected to have low contact resistance owing to the reduction of access resistance. As an extreme case, the lowest possible contact resistance that can be theoretically conceived is achievable in monolayer OTFTs^28,29^. Although this approach is promising, the metallization process in conjunction with subsequent patterning processes hampers the practical fabrication of monolayer OTFTs. It has been reported that the mobility of OTFT fabricated with vacuum evaporated gold contacts on a monolayer OSC film was two orders of magnitude lower than those of OTFTs fabricated with 2- or 3- molecular layers of OSC. To overcome this issue, development of a transfer or lamination process for contact electrodes, in which OSC layers are not exposed to the damage during vacuum evaporation, is a key challenge^30–34^. In the previous work^32^, the lamination technique has been demonstrated, where pre-patterned electrodes embedded into an elastic and flexible film, polydimethylsiloxane (PDMS), were employed. This method features scalability, compatibility with the photolithography process and excellent adhesion property onto various curved surfaces. However, in terms of environmental stability, PDMS, which has high thermal expansion coefficient, may cause some negative effects on monolayer OSC crystals because carrier transport layer is in proximity to PDMS.

Here, we demonstrate a simple and facile transfer method to deposit contact electrodes directly on the surface of OSC thin films. Use of a water-soluble polymer, polyvinyl alcohol (PVA), in conjunction with a thin buffer polymer, poly(methyl methacrylate) (PMMA), allows reliable, reproducible electrode transfer to any given destination substrate. OTFTs composed of monolayer single crystalline films fabricated with the present method exhibited a high mobility of >10 cm^2^ V^−1^ s^−1^ and a relatively low contact resistance of 175 Ω⋅cm.

## Results

### Transferring precisely patterned electrodes

**Figure 1:**
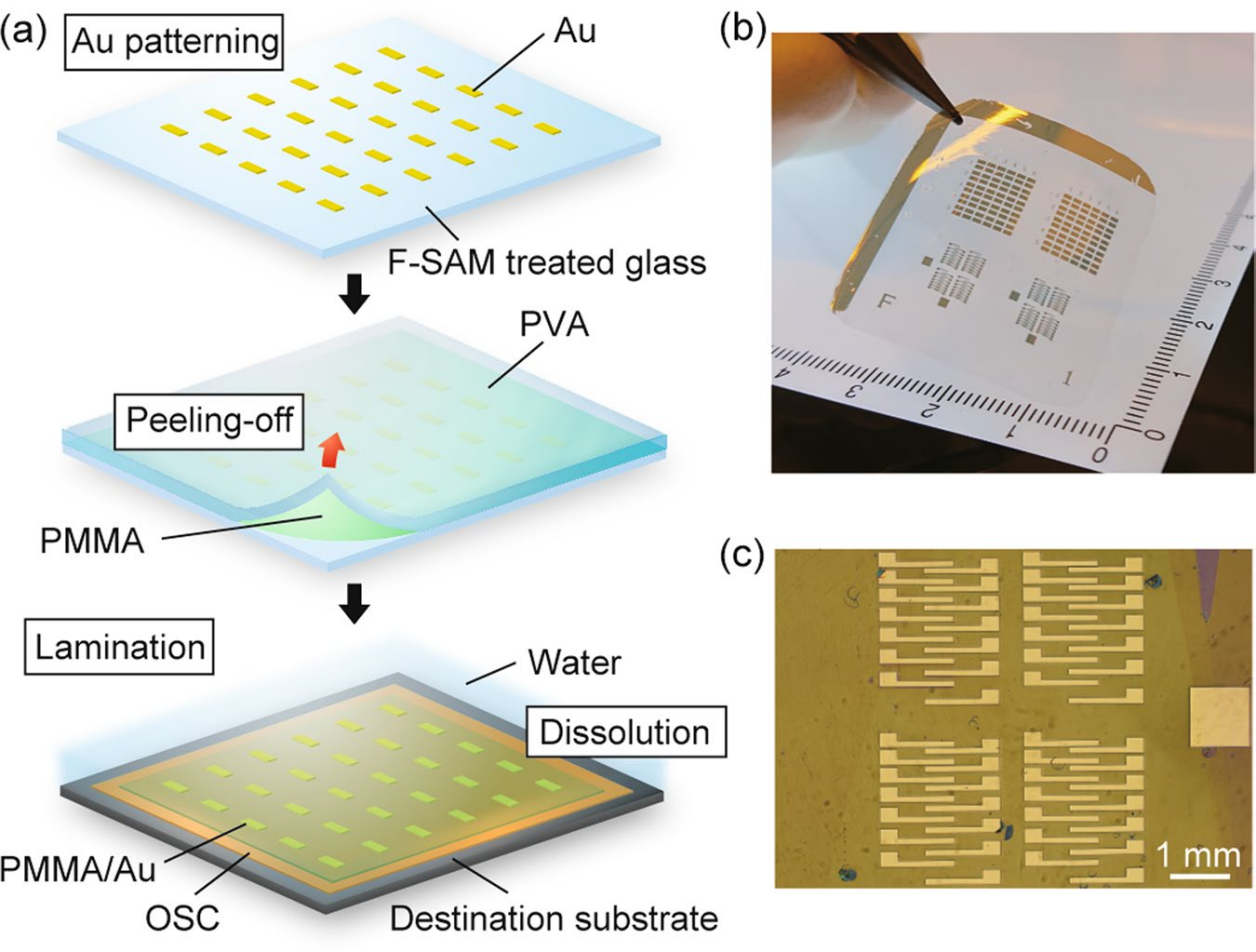
(**a**) Schematic illustration of the present transfer method. (**b**) Photograph of a PVA/PMMA/Au film exfoliated from the template substrate. (**c**) An optical microscopy image of the electrodes laminated on the destination substrate.

The key to the present electrode transfer method is the use of a polymeric bilayer film (PVA/PMMA) as a template. A 20– to 30–μm-thick PVA layer allows the whole template to be easily handled, and it was later dissolved in water easily (i.e., it is a sacrificial layer)^35–37^. A second polymer layer, a 100–nm-thick PMMA layer, helps the template adhere to a destination substrate. A schematic illustration of the present transfer method is shown in Fig. 1(a). First, a 40–nm-thick gold layer was deposited by vacuum evaporation on a glass substrate treated by a self-assembled monolayer (SAM) of triethoxy-1H,1H,2H,2H-heptadecafluorodecylsilane (F-SAM). To form the patterns for source and drain electrodes, a standard photolithography/metal etching process was performed. Subsequently, PMMA with an average molecular weight of 120,000 was spin-coated from 3 wt% butyl acetate solution to form a protective layer with a thickness of *ca*. 100 nm. Note that this protective layer needs to be thin enough to adhere to the destination substrate by electrostatic force. A sacrificial layer of 5 wt% aqueous solution of PVA was applied on the PMMA layer followed by drying at 50 °C for 2 h. The resulting PVA/PMMA/Au hybrid film had a thickness of 20–30 μm and could be easily peeled off from the glass substrate by adhesive tape [Fig. 1(b)]. Then, the hybrid film was placed on the surface of an OSC layer (destination substrate). Moderate heating at 80 °C was found to induce a controllable melt of the PVA/PMMA film, resulting in adhesion of the hybrid film onto the OSC layer. Finally, application of water removed the PVA layer, and the remaining PMMA/Au layer could be laminated electrostatically on the surface of OSC layer. The substrates were then cooled down to 30 °C and stirred in water to completely remove the residual PVA. Preservation of the fabricated patterns was confirmed by a polarized optical microscopy image of the electrodes laminated on the destination substrate, as shown in Fig. 1(c).

**Figure 2:**
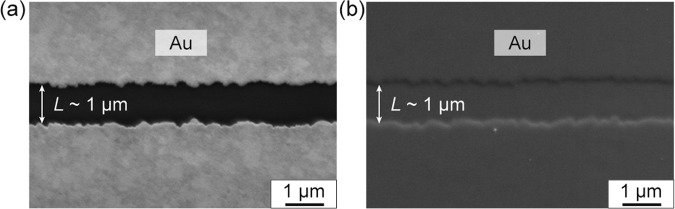
SEM images of (**a**) Au electrodes with a channel length of 1 μm formed on the F-SAM-treated glass substrate and (**b**) electrodes laminated on the destination substrate with the PMMA protective layer.

Because the present technique is compatible with photolithography, the spatial resolution of patterned metal electrodes can be reduced down to photolithography resolution (almost equivalent to the used wavelength). Figure 2(a,b) shows scanning electron microscopy (SEM) images of the electrodes patterned on a glass substrate treated with F-SAM and electrodes laminated on the destination substrate. Electrodes with a channel length of 1 μm were successfully transferred and laminated onto the destination substrate; neither expansion nor contraction of the gap was observed after the transfer was complete. Note that the spatial resolution of 1 μm is merely a limitation defined by our photolithography apparatus. We presume that the spatial resolution of the present transfer method can be improved by optimizing photolithography conditions, which will be future work.

### Fabrication and evaluation of monolayer OTFTs

**Figure 3:**
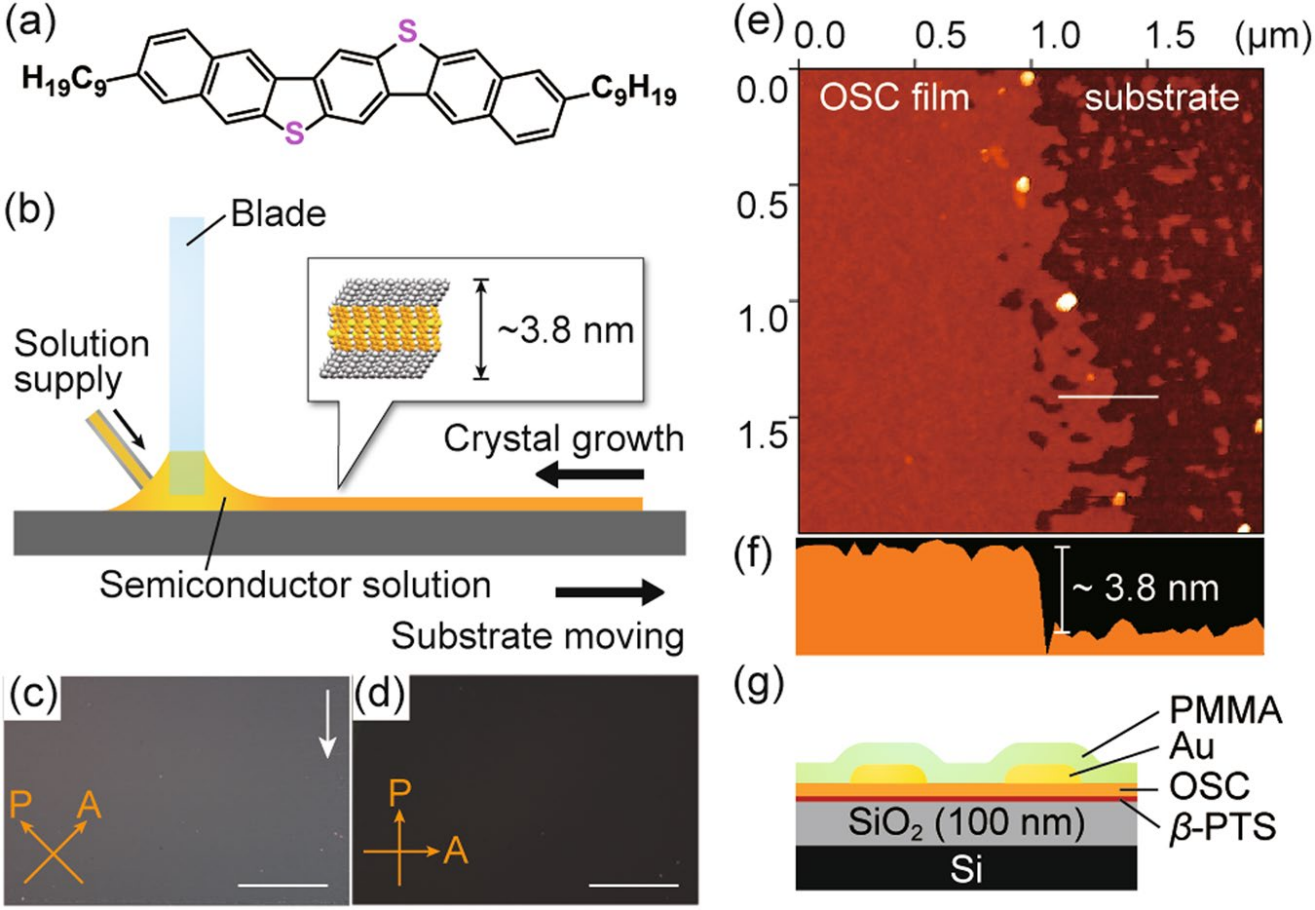
(**a**) Molecular structure of C_9_–DNBDT–NW. (**b**) Schematic illustration of the continuous edge-casting method. (**c**,**d**) Cross-polarized optical microscopy images of the fabricated monolayer single-crystalline film. (Scale bars: 250 μm.) The white arrow denotes the direction of crystal growth. (**e**) Atomic force microscopy image and (**f**) cross-sectional profile at the white line of the monolayer film. (**g**) Schematic illustration of the device configuration.

With the present method, contact electrodes are likely to be deposited directly on the OSC layer without any thermal damage or solvent damage. To verify this, we laminated Au electrodes on top of a monolayer OSC. A doped Si wafer with 100–nm-thick SiO_2_ was treated with trimethoxy(2-phenylethyl)silane (*β*-PTS) by vapor deposition. A single crystalline film of 3,11-dinonyldinaphtho[2,3-*d*:2′,3′-*d*′]benzo[1,2-*b*:4,5-*b*′]dithiophene [C_9_–DNBDT–NW, Fig. 3(a)], which is one of the DNBDT analogs^12,38^, was grown via a continuous edge-casting method^19,39–41^ [Fig. 3(b)]. By controlling the deposition conditions, such as the substrate temperature, solution supply rate, and substrate shearing, uniform thin films of C_9_–DNBDT–NW were deposited. Figure 3(c,d) shows cross-polarized optical microscopy images of the fabricated films. The formation of a highly ordered crystalline thin film is demonstrated by the simultaneous extinction of the obtained image as the crystal growth direction is positioned parallel or perpendicular to the polarization angle. The film thickness was estimated to be approximately 3.8 nm from atomic force microscopy measurements [Fig. 3(e,f)], which is equivalent to the height of the C_9_–DNBDT–NW monolayer film. After the deposition of contacts onto the monolayer films by the present transfer process, the OSC layer was electrically isolated using an yttrium-aluminum-garnet laser to avoid fringe currents^42^. For comparison, monolayer OTFT with vacuum evaporated gold contacts was also fabricated. A schematic illustration of the monolayer OTFT configuration is shown in Fig. 3(g).

**Figure 4:**
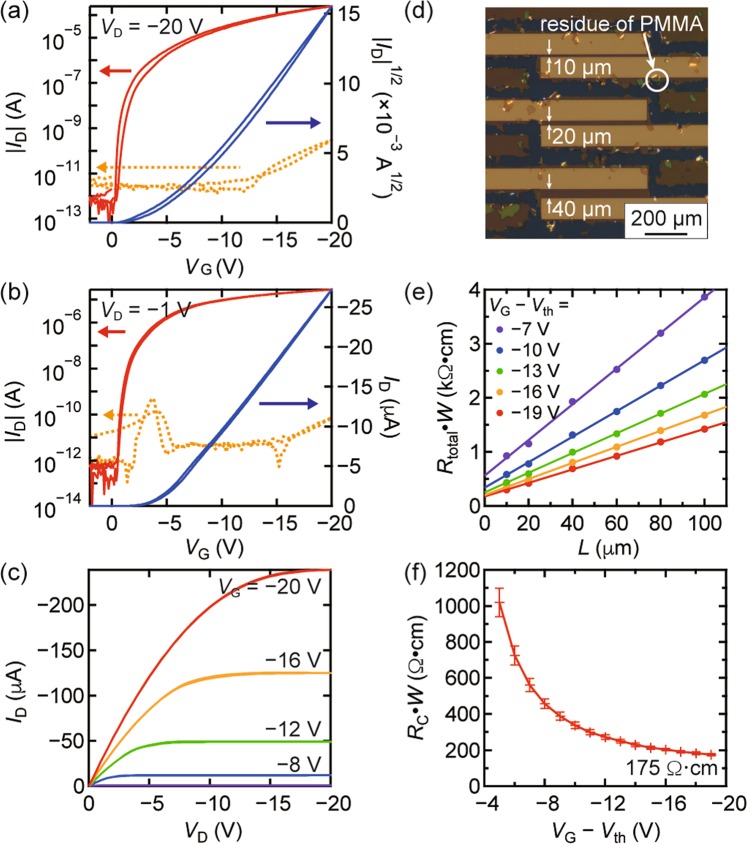
Transfer characteristics of monolayer OTFTs for (**a**) the saturation regime and (**b**) the linear regime. Solid lines denote transfer characteristics of a device with transferred gold electrodes (via the present technique), and dashed lines denote those with vacuum evaporated gold electrodes. (**c**) Output curves for the OTFT. The channel length (*L*) and width (*W*) are 200 μm and 1000 μm, respectively. (**d**) Cross-polarized optical microscopy image of three OTFTs for TLM measurement with *L* = 10 μm, 20 μm, and 40 μm and *W* = 500 μm. (**e**) TLM plots for monolayer OTFTs at various gate voltages. (**f**) Changes in the contact resistance as a function of the gate voltage for monolayer OTFTs.

The transistor characteristics of the monolayer OTFT are shown in Figs. 4(a–c). The mobilities extracted from the transfer characteristics were 12 cm^2^ V^−1^ s^−1^ and 10 cm^2^ V^−1^ s^−1^ in the saturation and linear regimes, respectively, while monolayer OTFT fabricated with vacuum evaporated gold contacts did not show transistor operation. Note that in the previous work^14^, OTFT with monolayer DNBDT analog did work even though the mobility was two orders of magnitude lower than those with multilayers. The difference can be attributed to the existence of thin carrier injection layer, 2,3,5,6-tetrafluoro-7,7,8,8-tetracyanoquionodimethane (F_4_-TCNQ), which may also serve as a buffer layer for the subsequent metal deposition. The value of the subthreshold swing (*S*) estimated from the transistor characteristics was less than 100 mV decade^−1^, which is much lower than that obtained for multilayers of DNBDT TFTs with the vacuum evaporated gold contacts^43^. Generally, *S* is associated with the density of deep traps located at the carrier transport interface or in the bulk of the semiconductor^44^. Because the combination of semiconductor/insulator is identical to that from the previous research, the low *S* is likely to be attributed to the damage-free interface between the metal and OSC layer achieved by the present transfer technique. The overall results suggest that the present electrode transfer method gives an ideal metal/OSC interface without any fatal damage, even to the monolayer film.

To assess the contact resistance (*R*_C_), several OTFTs with different channel lengths (*L*) of 10, 20, 40, 60, 80, and 100 μm were fabricated for monolayer single crystalline films, and *R*_C_ was estimated from the transmission line method (TLM) [Fig. 4(d)]. Prominent points observed in Fig. 4(d) come from the residue of the PMMA film after laser etching process. Fig. 4(e) shows the width-normalized total resistance (*R*_total_ ⋅ *W*) as a function of *L* with various *V*_G_ − *V*_th_ values (where *V*_G_ and *V*_th_ are the gate and threshold voltages, respectively). *R*_C_ can be extrapolated with high accuracy as the square of a regression coefficient (*R*^2^) higher than 0.99. The values of *R*_C_ ⋅ *W* for various *V*_G_ values are presented in Fig. 4(f). The monolayer OTFTs fabricated with the present method exhibited a relatively low *R*_C_ of 175 Ω⋅cm. Although *R*_C_ is higher than that reported for the previous work^14^, it can be improved by an introduction of molecular dopant layer, such as F_4_-TCNQ^45^. It should be noted that a wide variety of electrode materials other than evaporated gold can be employed for the present method.

We now turn to a discussion of why the metal lamination on the surface of an OSC provides an ideal interface for carrier injection. In general, it is difficult to control the quality of a heterointerface, particularly in covalent semiconductors like Si. This is because the Fermi level at the surface can be pinned by a large number of surface states that originate from surface dangling bonds (Bardeen limit). The Fermi level pinning effect can be a frustrating issue for designing semiconductor devices to some extent^46^. Unlike covalent semiconductors, there are essentially no active dangling bonds at the surface of organic semiconductor crystals because covalent bonds are in principle completed within each molecule. As evidence, ideal carrier injection has been realized by laminating molecular crystals having a molecularly flat surface, such as rubrene and tetracene, onto metal electrodes^47–49^.

Disorder-induced gap states can be another issue that needs to be optimized, because whether Schottky barrier heights at metal/semiconductor heterostructures are restricted by a Schottky limit or a Bardeen limit is thought to be dominated by the trap states at the interface. This is particularly problematic when fabricating top-contact OTFTs because OSC layers are susceptible to thermal damage during vacuum evaporation of metal electrodes. The present electrode transfer method can be an ideal approach to minimizing disorder-induced gap states. Concomitantly with the non-dangling bond nature of the C_9_–DNBDT–NW single crystalline monolayer film, Schottky-limited contact was realized in the present OTFT devices.

### Reproducibility of the transfer technique

**Figure 5:**
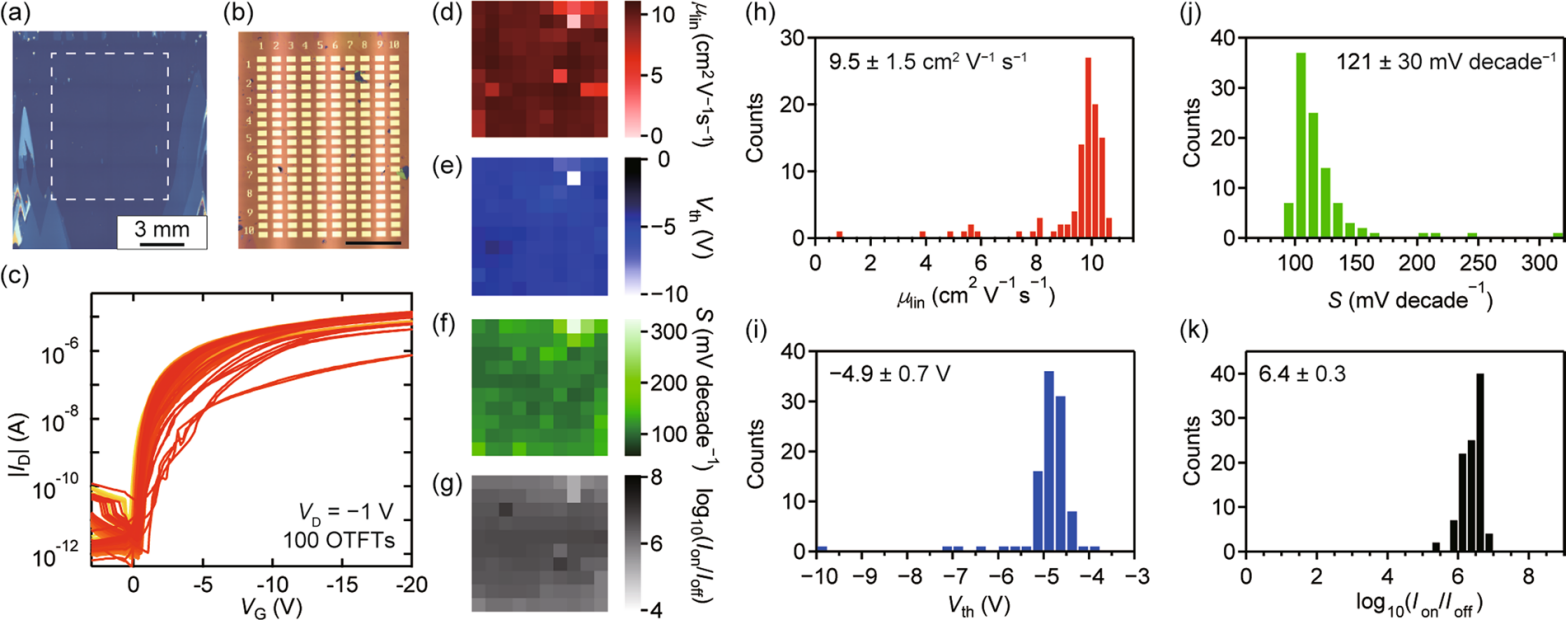
(**a**) Laser confocal microscopy image of the C_9_–DNBDT–NW film fabricated via continuous edge-casting method. The area within the dashed square was used for the investigation of device-to-device deviation. (**b**) Laser confocal microscopy image of a 10 × 10 OTFT array. (Scale bar: 3 mm). (**c**) Transfer characteristics of 100 OTFTs. *L* and *W* are 200 μm and 500 μm, respectively. Mappings of (**d**) *μ*_lin_, (**e**) *V*_th_, (**f**) *S*, and (**g**) ${{\rm{l}}{\rm{o}}{\rm{g}}}_{10}({I}_{{\rm{o}}{\rm{n}}}/{I}_{{\rm{o}}{\rm{ff}}})$. Histograms of (**h**) *μ*_lin_, (**i**) *V*_th_, (**j**) *S*, and (**k**) ${{\rm{l}}{\rm{o}}{\rm{g}}}_{10}({I}_{{\rm{o}}{\rm{n}}}/{I}_{{\rm{o}}{\rm{ff}}})$.

To assess reliability and reproducibility on the damage-free fabrication of monolayer OTFTs, a TFT array with monolayer C_9_–DNBDT–NW was fabricated and characterized. Figure 5(a) shows a laser confocal microscopy image of the C_9_–DNBDT–NW monolayer film fabricated on a SiO_2_/Si substrate treated with *β*-PTS. The differences in color contrast indicate differences in the thickness of films. A 10 × 10 OTFT array was fabricated within the area marked by the dashed square in Fig. 5(a), where the C_9_–DNBDT–NW monolayer film was formed [Fig. 5(b)]. All the 10 × 10 arrayed OTFTs showed transistor operation with an average linear mobility of 9.5 ± 1.5 cm^2^ V^−1^ s^−1^, which manifests the excellent reproducibility of the present transfer method [Fig. 5(c)]. The values of the mobility, *V*_th_, *S* and *I*_on_/*I*_off_ in the linear regime are summarized in Fig. 5(d–k). Note that some devices showed degraded performance. It is due to air bubbles which were generated when the PVA/PMMA/Au film were laminated on the OSC film. Although further investigation is needed, the process can be improved by optimizing the process conditions during lamination of electrode films onto the destination substrate with a suitable apparatus, and then it can be extended to more sophisticated mass-production methods, such as a roll-to-roll manufacturing.

## Discussion

In this study, we successfully developed a method to transfer contact electrodes onto ultrathin OSC films. The present method is compatible with photolithography, and allows precise deposition of electrodes with channel lengths as short as 1 μm without any expansion or contraction during the fabrication and transfer process. The key in this paper is to employ polymeric bilayer composed of thick PVA film and thin protective layer. Because the mechanism of the adhesion relies on the electrostatic force of the thin protective layer, the materials for this layer are limited not only to PMMA but also to other functional thin films such as encapsulation film or vertically stacked electrodes and insulating layer structures are also available. Contact electrodes can be deposited without any critical deterioration of the OSC layer. As a result, high-performance monolayer OTFTs can be successfully fabricated with a mobility higher than 10 cm^2^ V^−1^ s^−1^ and a relatively low *R*_C_ of 175 Ω⋅cm. The operation of all 100 OTFTs in a 10 × 10 array indicated that the present method can produce monolayer OTFTs reproducibly. The technique demonstrated here opens the door to a simple manufacturing approach, which is also useful for manufacturing other vertically stacked devices, such as organic solar cells and organic light-emitting diodes, and it will be extendable to roll-to-roll manufacturing in the future.

## Methods

### Preparation of Au/PMMA/PVA films

An EAGLE XG glass substrate (Corning Inc.) with a thickness of 0.7 mm was treated with UV/O_3_ for 15 min before F-SAM was deposited onto the surface by vapor deposition at 150 °C for 3 h. Subsequently, a 40–nm-thick gold layer was deposited on the glass by vacuum evaporation. Photolithography was used to form the pattern of the source and drain electrodes. A positive photoresist, AZ 5214 E (MicroChemicals), was spin-coated on top of the gold followed by baking on a hot plate at 105 °C for 1 min. After the substrate was exposed to UV light (*λ* = 375 nm) using an MLA 150 Maskless Aligner (Heidelberg Instruments) at a power of 100 mJ cm^−2^, it was developed in NMD-3 2.38 % (Tokyo Ohka Kogyo Co., Ltd.). The gold layer was patterned via a wet etching process using a gold etchant, AURUM S-50790 (Kanto Chemical Co., Inc.), followed by rinsing of the residual etchant with deionized water for 15 min. To remove the photoresist, the substrate was immersed in acetone. Then, PMMA with an average molecular weight of 120,000 (Sigma-Aldrich Co., LLC) was spin-coated from 3 wt% butyl acetate solution (1 s for acceleration, 500 rpm for 5 s, and 2,000 rpm for 30 s) as a protective layer on top of the patterned electrodes and then annealed on a hot plate at 80 °C for 30 min. An aqueous solution of 5 wt% PVA (average degree of polymerization of 1,500–1,800 with a saponification degree of 78–82 mol%, FUJIFILM Wako Pure Chemical Corp.) was applied on top of the PMMA layer and dried at 50 °C for 2 h. Finally, the resulting films were peeled off the glass substrate using adhesive tape.

### Fabrication of OSC layers

A doped Si wafer with thermally oxidized SiO_2_ (100 nm) was cleaned by sonication with acetone and 2-propanol for 10 min in each solvent. Subsequently, UV/O_3_ treatment was performed for 15 min before the SAM of *β*-PTS was deposited on the SiO_2_ surface by vapor deposition at 120 °C for 3 h. Then, the substrate was cleaned again with toluene and 2-propanol for 10 min in each solvent. Single-crystalline thin films of C_9_–DNBDT–NW were fabricated from 0.02 wt% 3-chlorothiophene solution via continuous edge casting^39^. To obtain large-area uniform thin films, the substrate was heated to around 84 °C and moved at a shearing speed of 22 μm s^−1^. The resulting films were dried at 80 °C in a vacuum oven to remove the residual solvents.

### Fabrication of OTFTs

The PVA/PMMA/Au hybrid films were mounted on the surface of C_9_–DNBDT–NW films with heating at 80 °C which resulted in the adhesion of the hybrid film onto the C_9_–DNBDT–NW layer. Then, deionized water was applied to dissolve the PVA layer. To completely remove the residual PVA, the substrates were cooled down to 30 °C and stirred in water. Finally, the PMMA/C_9_–DNBDT–NW films were patterned by a dry-etching process with a yttrium-aluminum-garnet laser (V-Technology Co., Ltd., Calisto, *λ* = 266 nm).

### SEM measurements

The SEM measurements were performed using a JSM-7800F Prime SEM (JEOL Ltd.) with an accelerating voltage of 1.5 kV at room temperature. The laminated electrodes were coated with 3–nm-thick platinum to reduce electron beam damage.

### Electrical measurements

A semiconductor parameter analyzer (Keithley 4200-SCS) was used to measure the electrical properties. All measurements were conducted in the dark under ambient air and room temperature. The mobilities in the saturation (*μ*_sat_) and linear (*μ*_lin_) regimes were extracted from the transfer characteristics using the following equations:

1 $$\begin{array}{rcl}{I}_{{\rm{D}},{\rm{s}}at} & = & \frac{{\mu }_{s{\rm{a}}{\rm{t}}}W{C}_{{\rm{i}}}}{2L}{({V}_{{\rm{G}}}-{V}_{{\rm{t}}{\rm{h}}})}^{2},\\ {I}_{{\rm{D}},{\rm{l}}{\rm{i}}n} & = & \frac{{\mu }_{{\rm{l}}{\rm{i}}{\rm{n}}}W{C}_{{\rm{i}}}}{L}{V}_{{\rm{D}}}({V}_{{\rm{G}}}-{V}_{{\rm{t}}{\rm{h}}}),\end{array}$$

where *I*_D_, *L*, *W*, *C*_i_, *V*_G_, *V*_th_, and *V*_D_ are the drain current, channel length, channel width, capacitance per unit area, gate voltage, threshold voltage, and drain voltage, respectively. A value of 34.5 nF cm^−2^ was used as *C*_i_ for all devices.

## Data Availability

The data that support the plots within this paper and the other findings of this study are available from the corresponding author (Shun Watanabe; swatanabe@edu.k.u-tokyo.ac.jp) upon request.

## References

[1] Gelinck GH (2004). Flexible active-matrix displays and shift registers based on solution-processed organic transistors. Nat. Mater..

[2] Yagi I (2008). A flexible full-color AMOLED display driven by OFETs. J. Soc. for Inf. Disp..

[3] Sekitani T (2009). Stretchable active-matrix organic light-emitting diode display using printable elastic conductors. Nat. Mater..

[4] Cantatore E (2006). A 13.56-MHz RFID system based on organic transponders. IEEE J. solid-state circuits.

[5] Myny K (2010). Organic RFID transponder chip with data rate compatible with electronic product coding. Org. Electron..

[6] Mannsfeld SC (2010). Highly sensitive flexible pressure sensors with microstructured rubber dielectric layers. Nat. Mater..

[7] Kaltenbrunner M (2013). An ultra-lightweight design for imperceptible plastic electronics. Nature.

[8] Ren X (2016). A low-operating-power and flexible active-matrix organic-transistor temperature-sensor array. Adv. Mater..

[9] Wang S (2018). Skin electronics from scalable fabrication of an intrinsically stretchable transistor array. Nature.

[10] Minemawari H (2011). Inkjet printing of single-crystal films. Nature.

[11] Nakayama K (2011). Patternable solution-crystallized organic transistors with high charge carrier mobility. Adv. Mater..

[12] Mitsui C (2014). High-performance solution-processable n-shaped organic semiconducting materials with stabilized crystal phase. Adv. Mater..

[13] Iino H, Usui T, Hanna J-I (2015). Liquid crystals for organic thin-film transistors. Nat. Commun..

[14] Yamamura A (2018). Wafer-scale, layer-controlled organic single crystals for high-speed circuit operation. Sci. Adv..

[15] Diao Y (2013). Solution coating of large-area organic semiconductor thin films with aligned single-crystalline domains. Nat. Mater..

[16] Briseno AL (2006). Patterning organic single-crystal transistor arrays. Nature.

[17] Someya T (2004). A large-area, flexible pressure sensor matrix with organic field-effect transistors for artificial skin applications. Proc. Natl. Acad. Sci..

[18] Dimitrakopoulos CD, Malenfant PR (2002). Organic thin film transistors for large area electronics. Adv. Mater..

[19] Kumagai S (2019). Scalable fabrication of organic single-crystalline wafers for reproducible TFT arrays. Sci. Rep..

[20] Dürr AC, Schreiber F, Kelsch M, Carstanjen HD, Dosch H (2002). Morphology and thermal stability of metal contacts on crystalline organic thin films. Adv. Mater..

[21] Dürr A (2003). Morphology and interdiffusion behavior of evaporated metal films on crystalline diindenoperylene thin films. J. Appl. Phys..

[22] Tang Q (2008). Micrometer-and nanometer-sized organic single-crystalline transistors. Adv. Mater..

[23] Wei Z (2019). Nature of vacuum-deposited electrode induced thermal irradiation damage on organic transistors. Appl. Surf. Sci..

[24] Nakayama K (2014). High-mobility organic transistors with wet-etch-patterned top electrodes: A novel patterning method for fine-pitch integration of organic devices. Adv. Mater. Interfaces.

[25] Yao Y, Zhang L, Leydecker T, Samori P (2018). Direct photolithography on molecular crystals for high performance organic optoelectronic devices. J. Am. Chem. Soc..

[26] Peng B, Huang S, Zhou Z, Chan PKL (2017). Solution-processed monolayer organic crystals for high-performance field-effect transistors and ultrasensitive gas sensors. Adv. Funct. Mater..

[27] Arai S, Inoue S, Hamai T, Kumai R, Hasegawa T (2018). Semiconductive single molecular bilayers realized using geometrical frustration. Adv. Mater..

[28] Jiang L (2019). Realizing low-voltage operating crystalline monolayer organic field-effect transistors with a low contact resistance. J. Mater. Chem. C.

[29] Peng, B., Lau, H. Y., Chen, M. & Chan, P. K. Realization of ohmic-contact and velocity saturation in organic field-effect transistors by crystallized monolayer. *arXiv preprint arXiv:1908.01032* (2019).

[30] Loo Y-L (2002). Soft, conformable electrical contacts for organic semiconductors: High-resolution plastic circuits by lamination. Proc. Natl. Acad. Sci..

[31] Carlson A, Bowen AM, Huang Y, Nuzzo RG, Rogers JA (2012). Transfer printing techniques for materials assembly and micro/nanodevice fabrication. Adv. Mater..

[32] Zhao X, Tong Y, Tang Q, Liu Y (2015). Wafer-scale coplanar electrodes for 3D conformal organic single-crystal circuits. Adv. Electron. Mater..

[33] Cui N (2019). A photolithographic stretchable transparent electrode for an all-solution-processed fully transparent conformal organic transistor array. J. Mater. Chem. C.

[34] Cui N (2019). Photolithography-compatible flexible electrodes conformed onto an organic bulk crystal for fabrication of large-scale organic transistor array. IEEE Electron Device Lett.

[35] Schaper CD (2003). Patterned transfer of metallic thin film nanostructures by water-soluble polymer templates. Nano Lett..

[36] Schaper CD, Miahnahri A (2004). Polyvinyl alcohol templates for low cost, high resolution, complex printing. J. Vac. Sci. & Technol. B: Microelectron. Nanometer Struct. Process. Meas. Phenom.

[37] Miyamoto A (2017). Inflammation-free, gas-permeable, lightweight, stretchable on-skin electronics with nanomeshes. Nat. Nanotechnol..

[38] Okamoto T (2019). Next-generation organic semiconductors driven by bent-shaped *π*-electron cores. Polym. J..

[39] Soeda J (2013). Inch-size solution-processed single-crystalline films of high-mobility organic semiconductors. Appl. Phys. Express.

[40] Tsurumi J (2017). Coexistence of ultra-long spin relaxation time and coherent charge transport in organic single-crystal semiconductors. Nat. Phys..

[41] Makita T (2019). High-performance, semiconducting membrane composed of ultrathin, single-crystal organic semiconductors. Proc. Natl. Acad. Sci..

[42] Pei K, Chen M, Zhou Z, Li H, Chan PKL (2019). Overestimation of carrier mobility in organic thin film transistors due to unaccounted fringe currents. ACS Appl. Electron. Mater.

[43] Makita T (2017). Spontaneously formed high-performance charge-transport layers of organic single-crystal semiconductors on precisely synthesized insulating polymers. Appl. Phys. Lett..

[44] Blülle B, Häusermann R, Batlogg B (2014). Approaching the trap-free limit in organic single-crystal field-effect transistors. Phys. Rev. Appl.

[45] Soeda J (2011). Solution-crystallized organic field-effect transistors with charge-acceptor layers: High-mobility and low-threshold-voltage operation in air. Adv. Mater..

[46] Kanagasekaran T, Shimotani H, Shimizu R, Hitosugi T, Tanigaki K (2017). A new electrode design for ambipolar injection in organic semiconductors. Nat. Commun..

[47] Takeya J (2003). Field-induced charge transport at the surface of pentacene single crystals: A method to study charge dynamics of two-dimensional electron systems in organic crystals. J. Appl. Phys..

[48] De Boer R, Klapwijk T, Morpurgo A (2003). Field-effect transistors on tetracene single crystals. Appl. Phys. Lett..

[49] Sundar VC (2004). Elastomeric transistor stamps: reversible probing of charge transport in organic crystals. Science.

